# COVID-19 in Human, Animal, and Environment: A Review

**DOI:** 10.3389/fvets.2020.00578

**Published:** 2020-09-04

**Authors:** Ayman A. Swelum, Manal E. Shafi, Najah M. Albaqami, Mohamed T. El-Saadony, Ahmed Elsify, Mohamed Abdo, Ayman E. Taha, Abdel-Moneim E. Abdel-Moneim, Naif A. Al-Gabri, Amer A. Almaiman, Abdullah Saleh Al-wajeeh, Vincenzo Tufarelli, Vito N. Staffa, Mohamed E. Abd El-Hack

**Affiliations:** ^1^Department of Animal Production, College of Food and Agriculture Sciences, King Saud University, Riyadh, Saudi Arabia; ^2^Department of Theriogenology, Faculty of Veterinary Medicine, Zagazig University, Zagazig, Egypt; ^3^Department of Biological Sciences, Zoology, King Abdulaziz University, Jeddah, Saudi Arabia; ^4^Department of Agricultural Microbiology, Faculty of Agriculture, Zagazig University, Zagazig, Egypt; ^5^Department of Animal Medicine and Infectious Diseases, Faculty of Veterinary Medicine, University of Sadat City, Sadat City, Egypt; ^6^Department of Anatomy and Embryology, Faculty of Veterinary Medicine, University of Sadat City, Sadat City, Egypt; ^7^Department of Animal Husbandry and Animal Wealth Development, Faculty of Veterinary Medicine, Alexandria University, Rasheed, Egypt; ^8^Biological Application Department, Nuclear Research Center, Atomic Energy Authority, Cairo, Egypt; ^9^Pathology Department, Faculty of Veterinary Medicine, Thamar University, Dhamar, Yemen; ^10^Laboratory of Regional Djibouti Livestock Quarantine, Abu Yasar International Est. 1999, Arta, Djibouti; ^11^Department of Applied Medical Sciences, Community College of Unaizah, Qassim University, Buraydah, Saudi Arabia; ^12^Anti-Doping Lab Qatar, Doha, Qatar; ^13^DETO—Section of Veterinary Science and Animal Production, University of Bari Aldo Moro, Bari, Italy; ^14^Department of Poultry, Faculty of Agriculture, Zagazig University, Zagazig, Egypt

**Keywords:** COVID-19, epidemiology, clinical studies, animals, human, environment

## Abstract

The medical authority in China, especially in Wuhan city, reported on December 2019 a large number of highly fatal, rapidly spreading viral pneumonia caused by an unknown coronavirus. The common history of all the patients was their visiting a Wuhan's whole food store, where live animals and seafood are sold. Irrespective of the efforts of the Chinese authorities, the virus spread rapidly all over the world by travelers, provoking widespread attention by the media and panic. Many previous coronavirus epidemics had been recorded, such as severe acute respiratory syndrome (SARS) and Middle East respiratory syndrome (MERS), and the recently newly discovered epidemic is named coronavirus disease of 2019 (COVID-19). This disease is caused by SARS Coronavirus-2 (SARS-CoV-2), and this virus is antigenically related to the SARS virus (SARS-CoV), which had been detected in 2002, depending on clinical, serological, and molecular findings. There is rapid competition among the researchers to discover the source of the virus, understand the mechanism of the disease development, establish treatment strategies, and determine the factors affecting the incidence of infection and severity of the disease, and focus on the production of a vaccine. Coronaviruses are a group of single-stranded, positive-sense RNA genome viruses; its genome length varies from 26 to 32 kb. Coronavirus causes mild to severe respiratory disorders. In December 2019, several cases of pneumonia of unknown causes were found in Wuhan city, which is located in the Hubei province in China. Chinese health authorities investigated the problem and found that a new virus caused such infection and, using next-generation sequencing, found the 2019 novel coronavirus (2019-nCoV). It has been transferred from humans to humans and animals to humans (zoonotic). Coronaviruses cause multiple respiratory problems, varying from common cold to severe infections such as SARS. General symptoms of infection include fatigue, cough, and breathing problems such as shortness of breath, as described by World Health Organization. Serious cases may result in pneumonia, renal failure, and even death. We address current information about the new SARS Coronavirus-2 as well as the COVID-19 disease caused by it in this review.

## Introduction

Coronaviruses can infect a wide range of hosts such as cattle, pigs, horses, turkeys, cats, rats, dogs, and humans. These viruses cause serious diseases in humans such as severe acute respiratory syndrome (SARS) and pneumonia and other mild diseases such as common cold as well as those affecting the gut. The first isolation of coronavirus took place in 1937 from birds infected with the infectious bronchitis virus, which is capable of devastating poultry stock. Coronaviruses are the cause of about 15–30% of common colds (1).

In December 2019, many of the coronavirus (2019-nCoV)-infected people in Wuhan developed acute respiratory problems. The virus was named novel coronavirus-infected pneumonia (NCIP) (2, 3). The main clinical manifestations were pyrexia, myalgia, fatigue, coughs, dyspnea, and pneumonia, which were confirmed by radiographic examination of the chest (3–5). It was reported that NCIP can be transmitted between humans as well as during the incubation period (6–8). In one hospital, 29% of the medical staff and 12% of then admitted patients not infected with NCIP were highly suspected to be infected and eligible for transmitting the infection (5). The NCIP has spread all over the world, and almost all countries recorded patients infected with NCIP (9–13). On 11 February 2020, the number of NCIP confirmed cases was 44,672, and the number of deaths was 1,023 in China. On 30th January 2020, the WHO has announced the flare-up of NCIP as a public health emergency of urgent importance. The average time between the inception of the illness to the development of dyspnea was 8 days, and the average period for the evolution of acute respiratory distress syndrome (ARDS) was 10.5 days among the NCIP cases admitted to the hospital (3). The percentage of ARDS development was from 20 to 29% (3, 5). Many cases were treated with oxygen therapy. Another variant of the oxygen treatments for severely ill patients was a high-flow nasal cannula (HFNC) (14). Nevertheless, no studies were available to support the best of our knowledge of the use of HFNC to treat hospitalized NCIP patients. Here, we try to record HFNC's impact on this community. Virological classification of coronaviruses showed that they are a member of the subfamily Coronavirinae located within the Coronaviridae family under the order of Nidovirales. Due to the presence of spikes on their surface, these viruses possess a crown-shape figure, hence the prefix *corona*, which means crown in Latin, and their naming as coronaviruses. Coronaviruses are divided into alpha, beta, gamma, and delta subgroups, according to their genomic structure. The first two groups infect only mammals, inducing respiratory disorders in humans and gastroenteritis in animals (15, 16).

Until December 2019, the number of known coronaviruses known to infect humans was six: HCoV-NL63, HCoV-229E, HCoV-OC43, and HKU1, which cause mild diseases in immunocompetent patients with common cold manifestations. While the other two viruses were the causative agent of the coronavirus pandemics in 2002 and 2012. The SARS epidemic in 2002 and 2003 had a 10% mortality ratio caused by SARS-CoV. The other epidemic was MERS caused by MERS-CoV in 2012 with a 37% mortality ratio.

During December 2019, a new beta coronavirus was found in China [named 2019 novel coronavirus (2019-nCov) at this time] and caused numerous cases of pneumonia especially in Wuhan city. After analyzing the genome of the new virus, it was found to be about 79.5% similar to the genetic structure of SARS-CoV that caused the SARS epidemic during 2002–2003 (2). Accordingly, the International Committee of Taxonomy of Viruses renamed this newly detected virus SARS-CoV-2 (17). On 30 December 2019, the Wuhan local medical authority released an epidemiology warning due to the recording of a large number of pneumonia cases during November and December 2019 with unknown etiology; all the infected people had a shared history of dealing with the wholesale seafood store. On the 9th of January 2020, Chinese investigators published the complete genomic sequence of the novel coronavirus, now called SARS-CoV-2 (18) in synchronization with the publication of several papers and reports about the virus' clinical manifestation, epidemiology, and treatment protocols (3, 4, 19–21). Moreover, several websites were set up to follow the updates on the outbreak and the numbers of new cases every hour (22). By the end of January 2020, COVID-19 had been considered as a worldwide emergency of general health by the WHO. This is the sixth alert from the WHO after the breakout of Ebola disease in the Democratic Republic of the Congo (2019), Zika (2016), West African Ebola breakout (2014), polio (2014), and H1N1 (2009). Finally, the WHO characterized COVID-19 as a pandemic on the 11th of March 2020 (23). Table 1 shows the comparison between the most fatal coronaviruses. This review highlights the latest available information about the potential origin of SARS-CoV-2, symptoms, infection transmission methods, factors affecting prevalence, and the roles of individuals and governments to control its spread. The latest contributions to finding functional vaccines and treatments have also been described.

**Table 1:** Comparison between the most fatal coronaviruses.

	**SARS-CoV^a^**	**MERS-CoV^b^**	**SARS-CoV-2**	
No. of Cases	Since 2002 8,098	Since 2012 2,494	Since 31 December as of the 21st of April 2020 2,529,094	Since 31 December as of the 30th of June 2020 10,436,890
Deaths	774	858	174,573	508,876
CFR (%)	9.56	34.4	6.9	4.9
Countries infected	26	27	195	195
Symptoms (%)^*^	Fever (99–100) Dry cough (29–75) Dyspnea (40–42) Diarrhea (20–25) Sore throat (13–25) Cases required ventilation support (14–20)	Fever (98) Dry cough (47) Dyspnea (55) Cases required ventilation support (80)	**Late December of 2019** **(****3****)** Fever (98) Dry cough (76) Dyspnea (55) Diarrhea (3) Cases required ventilation support (8)	
			**16–24 February 2020** **(****24****)** Fever (87.9) Dry cough (67.7) Fatigue (38.1) Sputum production (33.4) Dyspnea (18.6) Sore throat (13.9) Headache (13.6) Myalgia or arthralgia (14.8) Chills (11.4) Nausea or vomiting (5.0) Nasal congestion (4.8) Diarrhea (3.7) Hemoptysis (0.9) Conjunctival congestion (0.8)	
			**25 March 2020** **(****25****)** Fever (47) Dry or productive cough (25) Sore throat (16) General weakness (6) Pain (5)	

a *https://www.cdc.gov/sars/about/fs-sars.html*.

b *https://www.who.int/emergencies/mers-cov/en/*.

* *Differences in percentages of symptoms may be attributed to the sample size or case severity*.

*CFR, case-fatality rate*.

## Causative Agent

CoVs are a subfamily of a single-strand RNA; they are large and enveloped viruses. From its genera, beta, alpha, delta, and gamma, beta and alpha-CoVs can infect humans (26). The viral invasion to the host cell begins when the enveloping glycoprotein spike (S) attach to the dipeptidyl peptidase 4 (DPP4) and angiotensin-converting enzyme 2 (ACE2)'s cellular receptors for MERS-CoV and SARS-CoV, respectively (27). The genomic RNA of the virus is generated inside the cytoplasm, replicated, and then the genomic RNA binds to nucleocapsid proteins and glycoproteins envelope to form virion-containing vesicles. Following that, the virus is released outside the cell by fusion with the plasma membrane (28). By the 10th of January 2020, the SARS-CoV-2 genomic sequence was detected for the first time; it appeared as new identified form of beta-CoV, and the genetic identity between the sequenced samples obtained from the origin of the outbreak in Wuhan matches by more than 99.98%. Genetically, SARS-CoV-2 was reported to be more similar to SARS-CoV than MERS-CoV (19, 29, 30). By using transmission electron microscopy (TEM), the ultrastructure particles of SARS-CoV-2 were reported in the human airway epithelium (18). It was determined that human ACE2 is a receptor for SARS-CoV-2 and SARS-CoV (2, 19, 31). However, the SARS-CoV-2's S protein bond to human ACE2 is weaker than that of SARS-CoV, solidifying the theory that SARS-CoV-2 induces mild disease manifestations in patients than that of SARS-CoV (29). Besides, SARS-CoV-2 forms a new secreted protein encoded by *orf8* and short protein encoded by *orf3b*. It was suggested that the SARS-CoV-2 orf3b play a key role in pathogenicity of virus and block the *IFN*β expression, while the functional domain of orf8 still elucidated (7). On the other hand, by February 18, 2020, Zhou et al. (2) concluded the cryo-ultrastructure of the full-length human ACE2 in a complex with the amino acid transporter B^0^AT1 at a 2.9 Å resolution. They detected that the complex (that contains closed and open conformations) was formed as a dimer. In addition, the complex of ACE2-B^0^AT1 may unite with S proteins that confirmed CoV infection and recognition. B^0^AT1 could be considered as important therapeutic target for SARS- CoV-2 infection suppression.

**Figure 1:**
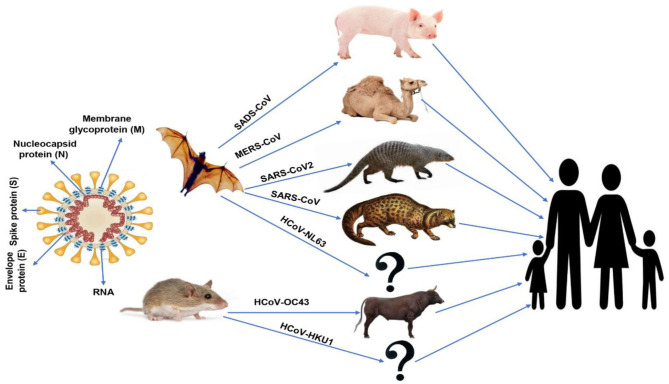
Schematic-labeled diagram of coronavirus (SARS-CoV) and animal origins of human coronaviruses.

The first identification of human coronaviruses (HCoV) occurred in the 1960s, as it was identified in the nostrils of patients with the common cold. There are two human coronaviruses, OC43 and 229E, which are responsible for a large proportion of common colds. Among humans, infection mostly occurs during winter and early spring. It is common for the instance of sickness to recur for the same person; one could become ill because of a coronavirus and then catch it again within 4 months later. This is possible because coronavirus antibodies do not last for a very long time. In addition, the antibodies for one strain of coronaviruses may be useless against other strains (1). All discovered coronaviruses that are causing illness for humans were originating from animals. Generally, these animals were either rodents or bats (32, 33). The spike proteins covering SARS-related coronaviruses contain many receptor-binding domains (RBD), which bind to angiotensin-converting enzyme-2 (ACE-2) receptor present in pneumocyte, cardiac cells, the gastrointestinal tract, and kidneys (34), which facilitates viral infection to the target cells. According to the phylogenetic analysis, the RBD of SARS-CoV-2 appears to be a mutated strain of its most closely related bat virus, RaTG13 (*Rhinolophus affinis*) (35). Due to this close relation, the SARS-CoV-2 was believed to infect people from bats after mutation. This mutation upregulated the RBD affinity to ACE-2 in humans and some animals like ferrets and Malayan pangolins (*Manis javanica*; an ant-eating mammal, which is illegally sold for traditional use in Chinese medicine), where it acts as intermediate hosts of SARS-CoV-2 (33), and decreased the affinity of RBD to ACE-2 found in civets and rodents. Little evidence indicated that SARS-CoV-2 originated from a manmade manipulation of an established coronavirus, but no supported evidence was present to support such hypothesis. In addition, Anderson et al. (35) indicate that the specific mutation, which was observed in the SARS-CoV-2 RBD, varies from what would have been expected based on genetic systems previously employed. However, scientists stated that “The other theories of (SARS-CoV-2) origin cannot currently be proved or disproved” (35). Bats were the source of both MERS-CoV and SARS-CoV that infect humans by civet cats and camels, respectively. Besides, bats were the natural hosts of SARS-CoV-2 as per the phylogenic studies of SARS-CoV-2 comparing with other CoVs, which showed that the novel virus is 96% identical to two SARS-like CoVs isolated from bats called bat-SL-CoVZX45 and bat-SL-CoVZX21 (2, 19, 29, 30). An intermediate host, which enables the novel virus to cross the species barrier to be able to infect humans, remains unknown. Ji et al. (36) suggested that snakes act as an intermediate host to the virus where homologous recombination within the S protein had occurred, transferring the virus from bats to humans. Another study in Guangzhou, China proposed that the ant-eating, long-snouted pangolins are the prospect intermediate host of SARS-CoV-2 depending on a 99% match in genetic identity between CoV discovered in pangolins and SARS-CoV-2 (37). A schematic-labeled diagram of coronavirus (SARS-CoV) and MERS-CoV and their transmission directly to humans from civet cats and dromedary camels, respectively, is illustrated in Figure 1.

## Method of Transmission of Infection

Transmission of human coronaviruses mainly occurs through air from an infected person to a healthy one by sneezing and coughing. Close physical contact seems to be transmitting the virus as well, such as rubbing or shaking hands and rubbing an object or surface contaminated with the virus and then touching one's lips, nose, or eyes before washing their hands. Transmission of theses virus rarely occurs through fecal contamination. Infection by human coronaviruses mainly occurs in fall and winter, bearing in mind that people can be infected by the virus at any time of the year and climate seems not to impede the transmission. Any person can be infected with one or more viruses of human coronaviruses during his life. The risk of being infected also extends to kids (1, 3, 20, 21).

The amount of the replication or “R naught” (R0) is a mathematical term describing contagiousness (38). It is the number of people that might be infected from one diseased host. If the value of R0 is <1, the illness is considered to be not highly contagious. If the value of R0 is 1 or more, then the disease will spread among humans. The SARS-CoV-2 R0 value estimates ranged from 2.24 to as high as 3.58 (39). The mean R0 for seasonal influenza for comparative purposes is between 1.1 and 2.3 (regional and immunization variables), while for SARS, it ranged from 1 to 2.75. The marginally higher R0 for SARS-CoV-2 may be as so because it has a longer prodromal period, which increases the time of contagion of the infected host. Coronaviruses are usually believed to be most easily transmitted by respiratory droplets, which is to not be confused with airborne infection (40). Droplets are of a large size and appear to fall to the ground near the infected host and infect others only if a susceptible host intercepts the droplet before landing. The transmission of droplets is normally limited to short distances, <2 m. The airborne infection, however, requires smaller droplets, which may float with air currents and traverse longer distances. Under specific temperature and humidity conditions, these airborne droplets can remain afloat for hours. Airborne pathogens usually have higher R0 values, since infected particles can still be airborne after the infected patient left the premises, as occurred in airborne measles infection (R0 12–18) (41) and chicken pox (R0s 3.7–5.0) (42). When the infected droplets fall on surfaces, their capacity for viability on those surfaces is the driving factor of the possibility of them being transferred by touch. According to available studies of other beta-coronaviruses, such as those on SARS and MERS, coronaviruses remain viable and infectious on inanimate surfaces such as glass, metal, or plastic from 2 h up to 9 days. Cold and dry conditions increase their virality (43–45); however, future investigations on SARS-CoV-2 are required to provide detailed information. Therefore, the Chinese government disinfects, and even sometimes destroys, cash as part of its attempts to suppress the virus (46). Fortunately, cleaning surfaces with sodium hypochlorite and ethanol or common biocidal substances is a very effective method to inactivate coronavirus within 1 min of exposure (45). To determine the period of optimal infectivity, a clinical study on 17 infected cases found that the nasal viral load peaked within days after the beginning of the clinical signs, indicating that disease transmission to another patient can occur early during infection (47).

## Incubation Period

A large number of people can be infected with SARS-CoV-2 simultaneously. Elderlies suffering from chronic diseases and pregnant women are highly susceptible to the infection (48). The infection varies according to the amount of exposure to the virus and the immune status of the host; a high infection dose of the virus with low immune status will increase the chance of infection and severity of the disease. Based on the report of the first 425 patients in China, the SARS-CoV-2 mean incubation period is 1–14 days, mainly 3–7 days (6). Another study on 1,099 patients reported that the incubation period ranged from 0 to 24 days with an average of 3 days (48). The most recent report based on a study on about 8,866 cases mentioned that the incubation period was 4.8 days (3.0–7.2) (49). Medical authorities must determine the effective quarantine period depending on the most accurate incubation period so that they can prevent infection by the virus during the incubation period (7). Understanding the duration of incubation is also necessary, as it helps health officials to implement more reliable quarantine schemes for critical events. The best present figures for contamination with SARS-CoV-2 range from 2 to 14 days. According to the aforementioned study, a median of 5.2 days incubation time was yielded (6). A later study, which was based on 1,324 cases, suggested a mean of 3 days as the incubation time (48). One study conducted between 20 and 28 January 2020 on 88 cases that traveled to Wuhan showed that the incubation time was between 2.1 and 11.1 days, with an average of 6.4 days (50). Individuals who were exposed to or diagnosed with the virus were typically expected to be quarantined for 14 days.

## Symptoms, Target Organs, Multiplication, and Body Response to Infection

Many coronaviruses, as types 229E, NL63, OC43, and HKU1, can infect humans and induce mild or moderate upper-respiratory problems such as the common cold. Such diseases last for a period, and most people get infected by these coronaviruses at any period of their life. The clinical signs of such illnesses are rhinorrhea, cough, pyrexia, and sore throat, with a common feeling of unwellness. In addition, human coronaviruses may induce lower-respiratory tract diseases such as bronchitis and/or pneumonia (1, 21). However, this is more prevalent in cases with chronic medical problems such as patients with cardiopulmonary disease and immunodeficiency patients, kids, and elderlies. MERS-CoV and SARS-CoV are the two coronaviruses that mainly cause severe diseases. The symptoms of MERS are high body temperature, cough, and difficulty in breathing that ends with pneumonia. Reports mention that out of every 10 MERS-infected patients, 3–4 people died. Cases of MERS primarily occur in the Arabian Peninsula. On the other hand, SARS clinical signs include high body temperature, chills, and body pain, which usually ended by pneumonia—although it is noteworthy that no cases of SARS were found all over the world since 2004 (1, 21).

The biochemical associations and pathogenesis of SARS-CoV-2 are definitely similar to SARS-CoV. Both bind to the receptors of angiotensin-converting enzyme-2 (ACE-2) in pneumocytes type II in the lungs, leading to lower respiratory tract inflammation (51). It was clear that binding of SARS spike protein to the ACE-2 receptors resulted in proteolytical processing of complex using (TMPRSS2) type II transmembrane protease leading to the cleavage of ACE-2 and the activation of spike protein (52, 53). This mechanism is similar to that used by viruses of influenza and metapneumovirus in humans, thus promoting the entrance of the virus inside the target cells. It has been suggested that cells, where both ACE-2 and TMPRSS2 are present simultaneously, are more vulnerable to SARS-CoV entry (54). Likewise, early reports mentioned that SARS-CoV-2 infection needs ACE-2 and TMPRSS2 to infect the target cells (2). Viral entry and cell invasion activate the immune response of the infected host and antigen-presenting cells (APCs) begin the inflammatory process. The cycle begins with the APC as they conduct two roles: (1) presenting the viral antigen to CD4+-T-helper (Th1) cells and (2) releasing interleukin-12 to further activate the Th1 cell (21). The Th1 cells stimulate CD8+-T-killer (Tk) cells, which attack any foreign antigen-containing cells. In addition, activated Th1 cells induce B cells to develop antibodies that are specific to the antigens. The frequencies of clinical signs recorded in the first clinical review were as the following percentages: fever, 98%; cough, 76%; and shortness of breath, 55% (3). Many cases showed less severe symptoms for 2–14 days before shortness of breath and severe signs appear. These patients still transmit the infection to whomever they come in contact with during this period and the disease course takes about 8 days. All patients admitted to the hospital suffered from clinical pneumonia, which was confirmed by CT scanning; about 32% of the patients showed hypoxia necessitating ICU admission. In addition, 10% needed ventilation, and two of them needed extracorporeal membrane oxygenation due to refractory hypoxia. The recorded case fatality rate (CFR) was 15%. Most of the dead patients suffered from comorbid conditions, and the average age was 49 as reported in the Chinese review (21). The most common and initial sign of COVID-19 is the fever, which it can pass with no complications. Otherwise, the patient will suffer from dry cough, bradypnea, myalgia, vertigo, headache, sore throat, runny nose, chest pain, diarrhea, nausea, and vomiting. After the onset of the disease by 1 week, some cases developed dyspnea and/or hypoxemia (48). In severely affected patients, they progress, some with an acute respiratory syndrome with septic shock, coagulopathy, and metabolic acidosis. Early diagnosis via viral detection must be conducted to patients suffering respiratory distress and acute fever, even without abnormalities in pulmonary imaging (4, 55, 56). In late December 2019, the demographic study reported that the percentages of the symptoms were 98% for fever, 76% dry cough, 55% dyspnea, and 3% diarrhea; 8% of the patients needed ventilation support (3). These percentages were confirmed by two recent investigations of a family cluster or a cluster infected from an asymptomatic individual (57, 58). A demographic investigation done in 2012 illustrated that individual who suffered from MERS had fever (98%), dyspnea (55%), and dry cough (47%) as their main signs, and 80% of them needed ventilation support; these values show that the patients in the MERS study where quite higher than that of patients who suffered from COVID-19. In addition, MERS had a higher lethality rate than that of COVID-19. Diarrhea and sore throat were also recorded with MERS patients at a rate of 26 and 21%, respectively. The frequency of clinical signs associated with SARS was recorded as 99–100% for fever, 29–75% for dry cough, 40–42% for dyspnea, 20–25% for diarrhea, and 13–25% for sore throat, and 14–20% required ventilation support (59). On 14 February 2020, the total confirmed cases of COVID-19 worldwide were 66,576 with a 2% mortality rate. While the total confirmed cases of SARS in November 2002 were 8,096 with a 10% mortality rate (60). In June 2012, the total confirmed cases of MERS were 2,494 with a 37% mortality rate (61). One study showed that the R0 of SARS-CoV-2 was higher than that of SARS-CoV; it was 6.47 for SARS-CoV-2 and ranged from two to four for SARS-CoV (20, 21, 62). Table 2 shows the deaths toll by age, gender, and underlying medical conditions in New York City, USA as of 14 April 2020.

**Table 2:** Deaths numbers by age, gender, and underlying medical conditions in New York City, USA as of 14 April 2020^b^.

	**Underlying conditions^a^**	**No underlying conditions**	**Unknown underlying conditions**	**Total**
Age				
0–17 years (%)	3 (0.04)	0	0	3 (0.04)
18–44 years (%)	244 (4.74)	25 (18.25)	40 (2.58)	309 (4.52)
45–64 years (%)	1,343 (26.07)	59 (43.07)	179 (11.53)	1,581 (23.11)
65–74 years (%)	1,272 (24.69)	26 (18.98)	385 (24.81)	1,683 (24.61)
≥75 years (%)	2,289 (44.44)	27 (19.71)	947 (61.02)	3,263 (47.71)
Unknown (%)	0	0	1 (0.06)	1 (0.01)
Gender				
Male (%)	1,873 (36.36)	37 (27.01)	620 (39.95)	2,530 (36.99)
Female (%)	3,087 (59.93)	96 (70.07)	912 (58.76)	4,095 (59.87)
Unknown (%)	191 (3.71)	4 (2.92)	20 (1.29)	215 (3.14)

a *Underlying illnesses include diabetes, lung disease, cancer, immunodeficiency, heart disease, hypertension, asthma, kidney disease, and GI/liver disease*.

b *https://www1.nyc.gov/assets/doh/downloads/pdf/imm/covid-19-daily-data-summary-deaths-04152020-1.pdf*.

## Mortality Rate and PM Findings

During the beginning of the COVID-19 pandemic, it was challenging to determine which citizens were extremely at hazard. Later, it turned out that the people who traveled to Wuhan city were extremely at an infection hazard, but there is no strict information for the citizens who are visiting the market. The Center for Disease Control and Prevention (CDC) in China released the epidemiological features of COVID-19 epidemics linked with the hazardous aspects of the mortality rate (40). It is known that the manner of the SARS-CoV virus development reflects that humans with an elevated average of ACE-2 receptors can be having an extensive hazard level. The titer of ACE-2 receptors can be associated with race, according to a study that proposed that White and African Americans had lower ACE-2-expressing ratios than the typical Asian male patient (39). Yet, the early study had only eight various persons (African Americans, Whites, and Asian), and it was concluded that these findings are unpractical.

In another report of 224 cases affected by bronchial carcinoma, ACE-2 receptor was expressed in tissues (63). Smoking history must be considered in characterizing the susceptible populations; ACE-2 gene expression was considerably increased in smokers. Since in China, smoking men are more than smoking women: 54% of men are smokers, while 2.6% of women are smokers (64). This justifies the remarkable gender difference presented in Chinese hospitals. The COVID-19 epidemic showed that children had a conserved class, yet this protection was due to that they were less likely to visit the Wuhan wet market and they have no symptoms or mild illness, and thus have not been examined. COVID-19 has infected 1-month-old babies (65), most with mild or no symptoms. Female individuals who were infected with COVID-19 during the gestation period did not transmit the disease to their infants. About 1,716 Chinese healthcare employees were affected with the coronavirus; five of them died. This terrible infection happened on the 17th of February 2020 (66). Table 3 shows patients, deaths, and fatality rate by age, gender, and underlying medical conditions for *n* = 44,672 confirmed COVID-19 cases in Mainland China as of February 11, 2020.

**Table 3:** Patients, deaths, and fatality rate by age, gender, and underlying medical conditions for *n* = 44,672 confirmed COVID-19 cases in Mainland China as of February 11, 2020^b^.

	**No. of cases**	**Deaths**	**CFR (%)**
Age			
0–19 years (%)	965 (2.16)	1 (0.10)	0.10
20–39 years (%)	11,219 (25.11)	25 (2.44)	0.22
40–59 years (%)	18,579 (41.59)	168 (16.42)	0.90
60–69 years (%)	8,583 (19.21)	309 (30.21)	3.60
≥70 years (%)	5,326 (11.92)	520 (50.83)	9.76
Overall	44,672	1,023	2.29
Gender			
Male (%)	22,981 (51.44)	653 (63.83)	2.84
Female (%)	21,691 (48.56)	370 (36.17)	1.71
^a^Comorbid Medical Conditions			
Hypertension	2,683 (12.89)	161 (31.94)	6.00
Diabetes	1,102 (5.30)	80 (15.87)	7.26
Cardiovascular Disease	873 (4.20)	92 (17.86)	10.54
Chronic Respiratory disease	511 (2.46)	32 (6.35)	6.26
Cancer	107 (0.51)	6 (1.19)	5.61
Non-comorbid Condition	15,536 (74.65)	133 (26.38)	0.86
Missing	23,690 (53.03)	617 (60.31)	2.61

a *The comorbid condition variable only includes a total of 20,812 patients and 504 deaths, and these values were used to calculate percentages in the confirmed cases and deaths columns*.

*CFR, case-fatality rate*.

b *http://weekly.chinacdc.cn/en/article/id/e53946e2-c6c4-41e9-9a9b-fea8db1a8f51*.

## Factors Affecting the Spreading and Increased of Mortality of the Virus

The WHO believes that coronavirus carriers are infectious 2 days before the onset of the symptoms (23). We, therefore, use 3-day average temperature and relative humidity up to and including the day when the R value is measured, respectively. The mortality rate of pulmonary diseases was elevated and strongly linked with the decreasing temperature (67–69). However, another report clarified that cold, as well as heat, can harm the pulmonary mortality rate (70). Moreover, a concluded report within 30 East Asian countries clarified that the higher the diurnal temperature range (DTR), the higher the mortality risk for pulmonary and cardiovascular affection present (71). In the cold atmosphere, the accumulative hazards of pulmonary and cardiovascular mortality grew with higher DTR rates (72). A time experiment conducted in Shanghai on the influence of DTR on chronic obstructive pulmonary disease (COPD) mortality clarified that, for every 1°C increase in the 4 days for DTR, COPD mortality increases by 1.25% (73). In an *in vitro* report on the effect of cold on immunity, Luo et al. (74) explained that due to the reduction in the phagocytic ability of macrophages present in alveolus beneath the cold temperature, cold impairs immunity functions (74). Cold air respiration enhances the constriction of bronchus and in turn promotes the tendency for pulmonary infection (75). Furthermore, SARS-CoV-2 struggles to survive in high-temperature settings besides other factors crucial for virus transmission, such as overcrowding and ill ventilation with winter seasons (76). In addition, the decrease in lung functions and the increase in aggravations for COPD patients were associated with cold temperatures (77). Besides, DTR considers a fixed temperature parameter, which is a key to variability of temperature to estimate influences on human health, including morbidity and mortality (78). Furthermore, abrupt variations of temperature add to the burdens of the respiratory and cardiac systems causing high levels of DTR and cardiopulmonary symptoms (72). Scientists reported that cold and low humidity conditions elevate the possibility of respiratory infection (79), where humidity was the principal key to mortality. Decreasing the humidity degree may cause higher mortality rates, most likely by influenza-related mechanisms (80, 81). Some reports also indicated that COVID-19 mortality decreased only with higher absolute humidity (82). In addition, the expansion of the influenza pandemic virus is very effective under dry and cold atmosphere (83), and the lesser the absolute humidity, the higher the survival rate of the influenza virus (84) that could be similar to the coronavirus. Thus, the elevation of COVID-19 mortality could also be associated to low humidity levels in winter. Nevertheless, it is worth noting that the previous expectations were during the first 3 months of the commencement of the pandemic, but now and after 6 months, it is not clear that the virus is less infectious during the summer. For example, the countries of southern Europe and the countries of the Middle East, which are now entering summer, are not assisting to decreases in infection rate, and this is probably due to less restriction to circulation and confinement. But at the same time, it appears that, in these circumstances, if no measures are considered, the virus has the potential to spread and infect a high number of people.

## Control of the Spreading of the Virus

Raj et al. (27) confirmed that coughing, sneezing, and materials contaminated with SARS-CoV-2 could be highly contagious with the disease. Feces also were found to be contaminated with the virus, which develops an unprecedented chance of feces–mouth transmission. An early report on 138 patients described that 41% of the patients probably were infected by nosocomial infections. Of these patients, 40 of them were healthcare employees and 17 were having previous illnesses (5). Thus, extreme vigilance is imperative for saving populations that communicate with patients or infected people.

Using disinfectants such as soap and alcohol-based antiseptics is effective in controlling viral spreading. Soap is a surfactant compound that has a hydrophilic head and a hydrophobic tail. Thus, when the lipid viral membrane encounter the amphipathic soap molecules, the hydrophilic head is attracted to water while the hydrophobic tail sticks to the lipid membrane, so the viruses are lifted off and washed away. As viruses become displaced, more soap molecules surround and destroy them by breaking open the lipid membrane of the virus. Alcoholic-based disinfectants also disrupt the lipid viral membrane, but in a different way from soaps, where most of these disinfectants contain either ethanol or isopropanol or a mixture between them dissolved in water. These alcohols are small polar molecules that can interact with the surface of the lipid layer and subsequently disrupt the viral membrane structure and breaking open the virus when alcohols are present in sufficient concentrations. Furthermore, denaturation of viral proteins is another mechanism of alcohol-based antiseptics to disrupt proteins structure on the surface of SARS-CoV-2 viruses and inactivate them. Alcohol inactivates the virus by displacing the hydrogen bonds between amino acids that maintain the shape of viral proteins, causing the loss of function and structure of these proteins. It is worth noting that it is difficult to disrupt viral proteins by this method in the absence of water; therefore, 60–80% alcohol products are most effective than those of 100%.

## Individual Roles

Using face masks represent one of the early steps of protection to reduce the infection hazard. Both surgical masks and N95 respirator masks (series # 1860s) could prevent infection (85). The runny droplets from the infected cases can pass through the atmosphere or hold onto materials' surfaces. This can be prevented by wearing surgical face masks (86). However, inhalation of virions (10–80 nm) can be protected only by N95 (series # 1860s) masks, with 5% of the virions capable of sneaking; SARS-CoV-2 is identical to SARS-CoV in size with an average of 85 nm (86). Thus, healthcare employees who communicate with infected cases should wear N95 (series # 1860s) masks and avoid surgical masks (87). Moreover, healthcare employees must have adequate medical clothes for protection against viruses. On January 22, 2020, a physician contracted SARS-CoV-2 despite wearing an N95 mask; SARS-CoV-2 could enter the body via inflamed eyes. Thus, viruses can also be transmitted through the eyes (19). It is strongly recommended that healthcare employees wear clear face shields or goggles during handling infected patients. For the infected regions, it is strongly recommended all people wash their hands with suitable disinfectant several times (personal cleanliness), stay indoors, and reduce direct contact with infected patients. Three feet is considered a recommended distance that is prompt for communicating with a patient (88).

## Governmental Roles

SARS-CoV-2 is considered a novel viral infection to the world and happens to be identical to SARS-CoV as announced last January 2020 (89). It caused a massive uproar in China as it was a throwback to the SARS outbreak in 2003. However, the government reassured people through spreading awareness about how to decrease contagiousness and lower infectivity from human to human to avoid an outbreak, easing the distress of the citizens during the spring festival. Later on, the disease control agencies in China conducted more definitive refinements such as the following: (1) checking information with the utmost scrutiny when notifying the public, as this affects the public attitude and decisions of citizens, (2) more reactivity to peculiar information from clinics, (3) higher restrain to still the epidemic at its early stage, and (4) elevate the public's awareness about pandemic diseases and upgrade the response system of the society periodically (90). On March 24, 2020, in Germany, the situation of epidemic situation of national relevance has been declared; the Federal Ministry of Health was empowered to adopt measures for the protection of the population and ensure the provision of healthcare, including (1) measures regarding cross-border transport such as reporting duties on the train or bus transport, (2) reporting and investigation obligations, (3) measures to ensure the basic provision of medicines, protective equipment, and laboratory diagnostics, (4) granting exceptions to the rules in medical and care institutions. The government also plays an efficient role in elevating awareness of population about pandemic diseases and the importance of social distancing and individual self-protection roles. On April 14, 2020, WHO highlighted general government strategies to respond to COVID-19 including the following: (1) lead and organize response across party lines to encourage and motivate all individuals and communities to take ownership of the response through communication, education, participation, capacity building, and support; (2) reuse and engage all available resource in the public community and private sector in a rabid manner up the public health system to end and track, isolate, and care for reported cases (whether at home or in a medical facility), locate, trace, quarantine, and assist contacts; (3) effectively help the health system to handle COVID-19 patient and sustain other important health and social services for everyone; and (4) enforce robust physical distancing steps and restrictions proportionate to the health risks faced by the community if necessary.

## Vaccination

Functional vaccines are in dire need to interrupt the transmission chain between animal carriers and affected people to sensitive hosts, which should be usually compatible with antiviral treatment in epidemics control. Several attempts were conducted to produce S protein-based vaccines against SARS-CoV for long-term immunity (91, 92). Live-attenuated vaccines have been studied in animal models (93). Nevertheless, the performance of these vaccine candidates cannot be established yet with their immunity to viral zoonotic infection until a clinical trial is conducted on elderly people and lethal-challenge models. This is because, until now, 17 years, SARS had no new event. Intermingle, on the other side, throughout the Middle East, sporadic cases, and fragmented of MERS has been established by the use of inactivated viruses, viral vectors, virus-like particles, DNA plasmids nanoparticles, and recombinant protein subunits that were assessed in animal models (94). The presence of a secure and successful vaccine for SARS-CoV-2 in non-immune populations is an imperious as well as definitive aim for preventing the viral spreading of Coronavirus. However, the timing can prove to be challenging due to the long period (18 months approximately) required for vaccine synthesis, testing, and creating mutable variations of CoVs (90).

## Vaccine Development

The target of SARS-CoV-2 research is potentially needed to develop a functional vaccine to neutralize SARS-CoV-2 antibodies. Several healthcare institutes in the world are researching on a vaccine development using their coronavirus background. Besides, the recent modalities of the SARS-CoV-2 spike protein can facilitate a semblance of a suitable vaccine (95). RNA vaccines can reduce efficient immunity against several infectious diseases and certain cancers (96, 97).

Conventional vaccines (live, attenuated vaccines) could activate the synthesis of antibodies. However, several years are required to produce highly functional vaccines. On the other hand, RNA-based vaccines utilize messenger RNA (mRNA), which is translated to antigenic molecules during entering the cells. Thus, this encourages the immunity. Such a theory was developed efficaciously for the treatment of some cancers (98, 99), and clinical experiences are underway for many other cancers (100). Furthermore, the development of RNA-based vaccines is characterized by rapidity and feasibility as well as considered as a great benefit to combat global infection. Several experiments for a SARS-CoV-2 vaccine (mRNA-based) are actually under research. Researches may draw the mRNA vaccine in double doses within a 28-day interval (21).

## Treatment

There are no specific treatments for illnesses caused by human coronaviruses. Most people with common human coronavirus illness will recover on their own. The present best treatment plan for COVID-19 is strictly for symptomatic such as the following: administration of medications for pain and fever, taking hot showers to console sore throat and cough, hydrating, and staying home and resting (1) During the 2003 SARS epidemic, physicians and intensive care specialists learned more strategies that enabled them to apply their information as a guide for the current medication situation of COVID-19. Currently, from this information, some are applied and utilized, such as admission to intensive care units when recommended, adhering to instructions for preventing the infection, and prevention of nosocomial transmission (101). However, several strategies were reported to enhance functional treatments. The major efficacious approach for COVID-19 treatment is evaluating the functional efficacy of the current antiviral drugs. In the past beta coronavirus epidemics, different medications, such as lopinavir-ritonavir, ribavirin, interferon, and darunavir/cobicistat (prezcobix) were *in vitro* examined with highly potential outcomes (102). Remdesivir, an adenosine analog utilizes resistant to RNA-coded viruses (SARS and MERS-CoV), was an *in vitro* candidate for Ebola treatment with great potential outcomes contrary to *in vivo* Ebola treatment (103, 104). Remdesivir was highly functional *in vitro* as it dominated over SARS-CoV-2 infection (5). Ameliorative usage of Remdesivir was utilized in the first COVID-19 case treatment in the United States; there was a spectacular amelioration for the clinical condition at quick clinical retrogradation (9). Unpredictable double-blinded clinical experiments are currently under research in China and the USA for estimating the effectiveness of Remdesivir, and primary outcomes should be predictable by the end of April 2020 (105). Other existent drugs comprise of Chloroquine and Camostat mesylate. Chloroquine is extensively applied as an antimalarial drug, which stops virus–cell fusion and halts glycosylation of SARS-CoV and ACE-2 cellular receptors, making the ACE-2–SARS-CoV interaction less functional (106). Furthermore, chloroquine showed promise *in vitro* evidence for inhibition of SARS-CoV-2 cellular entrance (5). In Japan, Camostat mesylate, also known as FOY 305 (107), was originally advanced and confirmed for the medication of chronic pancreatitis (108, 109). Camostat mesylate aims at the TMPRSS2 protease, which, theoretically, means the inhibition of the virus' entrance. Deutsch researchers confirmed that Camostat mesylate minimized the replication of SARS-CoV-2 (110). The uses of serum or convalescent plasma from recovered infected cases to treat patients represent an effective treatment modality. The treated patients from the viral infection produced a specific antibody as a response to SARS-CoV-2. Such antibody was helpful in the immunization against viruses in freshly infected patients. This strategy has been effectively utilized through the 2014–2015 Ebola outbreak (111, 112). However, the employ of convalescent sera has a restricted advantage in pandemic cases because the rapid expansion of affected cases overruns the donor plasma. The fresh reports confirmed that ACE-2 receptors were the target for both SARS-CoV-2 as well as 2002 SARS-CoV (2). These results increase the chances of using the previous studies on the 2002 SARS epidemic to COVID-19. The initial modality would be to use either a small RBD or an equalizing antibody targeting the ACE-2 receptor, thus prohibiting the binding of the S protein and inhibiting the virus entry into cells (113, 114). Definite monoclonal antibodies are considered as a powerful treatment method (115, 116). A specific time window must be given during the usage of RBDs or other antibodies in the treatment before the initiation of viral replication (51). Moreover, the adverse effects of ACE-2 blockade were comprehended. ACE-2 is widely distributed other than respiratory tissue and diminished prior application. Eventually, the viability of ACE-2 receptors affects the curative dose of RBD or the administered antibody. Another design for making an ACE-2-like molecule could fasten the coronavirus S protein itself. Once more, *in vitro* research on the 2002 SARS virus explained that the SARS virus can be blocked from infecting cells by soluble ACE-2 proteins (114, 117). The further advantage of using this modality is a possible inhibition of S-protein-mediated ACE-2 shedding, which offered the characteristic feature of SARS pulmonary edema (118, 119). A phase II clinical trial of recombinant ACE-2 in ARDS described a respectable modulation of inflammation; however, it is not in accordance with respiratory parameters (120). Additional studies are requested to evaluate the efficacy of animal studies to be of clinical benefit. Nowadays, more than 80 clinical attempts were performed to evaluate the potential SARS-CoV-2 treatments (121). These medications cover present and prospect treatments with antiviral therapeutics, immunosuppressive drugs, steroids, and recovered patients' plasma, psychological support, and traditional Chinese medications. Pharmaceutical companies are in a race to produce appropriate vaccines against the virus (90).

## Conclusion and Perspectives

The novel coronaviru' point of inception was from the seafood market at Wuhan, China where wild animals are sold, and are currently rapidly expanding all over the world. However, the provenance of SARS-CoV-2 is still unconfirmed. It is suggested that bats are the key to infection based on the sequence analysis. DNA recombination has been found to involve spike glycoprotein in the dispersed SARS-CoV (CoVZXC21 or CoVZC45) with the RBD of another beta CoV; therefore, this could be the cause for transmission and rapid infection. In the phylogenetic trees, SARS-CoV is similar to SARS-like bat CoVs. Currently, there is no appropriate clinical therapy or prevention methods used against human coronaviruses. Nevertheless, scientists are working hard to establish a medicine that can be used against novel coronaviruses. Different broad-spectrum antiviral drugs that were previously used against influenza, SARS, and MERS coronaviruses are suggested to be used either alone or in combinations to treat patients suffered COVID-19, clinical isolates, and mice models. In infected patients, Remdesivir, Oseltamivir, Lopinavir, and Ritonavir substantially blocked the infection with COVID-19. It can be inferred that the homologous recombination event at the RBD region's S protein improved the virus' transmission ability. Until now, the decision to return natives from the polluted region to homelands by different countries and the poor examination of passengers are the leading cause for spreading the virus in other countries.

## Author Contributions

All authors listed have made a substantial, direct and intellectual contribution to the work, and approved it for publication.

## Conflict of Interest

The authors declare that the research was conducted in the absence of any commercial or financial relationships that could be construed as a potential conflict of interest. The reviewer AS declared a past co-authorship with one of the authors VT to the handling editor.
